# Laterality and Flight: Concurrent Tests of Side-Bias and Optimality in Flying Tree Swallows

**DOI:** 10.1371/journal.pone.0001748

**Published:** 2008-03-12

**Authors:** James T. Mandel, John M. Ratcliffe, David J. Cerasale, David W. Winkler

**Affiliations:** 1 Department of Ecology and Evolutionary Biology, Cornell University, Ithaca, New York, United States of America; 2 Department of Neurobiology and Behavior, Cornell University, Ithaca, New York, United States of America; Smithsonian Institution, United States of America

## Abstract

Behavioural side-bias occurs in many vertebrates, including birds as a result of hemispheric specialization and can be advantageous by improving response times to sudden stimuli and efficiency in multi-tasking. However, behavioural side-bias can lead to morphological asymmetries resulting in reduced performance for specific activities. For flying animals, wing asymmetry is particularly costly and it is unclear if behavioural side-biases will be expressed in flight; the benefits of quick response time afforded by side-biases must be balanced against the costs of less efficient flight due to the morphological asymmetry side-biases may incur. Thus, competing constraints could lead to context-dependent expression or suppression of side-bias in flight. In repeated flight trials through an outdoor tunnel with obstacles, tree swallows (*Tachycineta bicolor*) preferred larger openings, but we did not detect either individual or population-level side-biases. Thus, while observed behavioural side-biases during substrate-foraging and copulation are common in birds, we did not see such side-bias expressed in obstacle avoidance behaviour in flight. This finding highlights the importance of behavioural context for investigations of side-bias and hemispheric laterality and suggests both proximate and ultimate trade-offs between species-specific cognitive ecology and flight biomechanics.

## Introduction

Hemispheric specialization, the division of neural processing tasks between the left and right hemispheres of the brain, is generally agreed to be responsible for sensoribehavioral side-biases in reptiles, birds, and mammals [1]–[3]. Hemispheric specialization and resultant perceptual side biases may provide animals with a hard-wired rubric for life-preserving decisions. One hypothesis suggests that lateralization of cognitive and visual processing minimizes response time (e.g., light-exposed chicks, *Gallus gallus domesticus*, always use the left eye for predator recognition when given a choice, and show longer habituation times to visual patterns when forced to use the right eye [1], [4]). In three species of toads (*Bufo* spp.) side-bias is expressed when individuals are confronted with predators; escape responses are faster when predator models are introduced from their left side than from their right, and the type of response (sideways vs. forward jumps) varies with side of presentation [5]. Hemispheric specialization, and associated perceptual biases and asymmetrical motor responses, appears to be highly conserved in vertebrates [6]–[7].

In birds, chicks [4], [8]–[9], pigeons, *Columba livia* [9]–[11], and black-winged stilts, *Himantopus himantopus* [12] have been shown to favour one hemisphere over the other for making specific decisions, including those involved in copulation and foraging. Whatever the underlying mechanisms, brain lateralization is positively correlated with efficient neural processing and multitasking [1], [3], [13]. Selection for such decision-making should lead to quicker response times, and might explain the apparent ubiquity of hemispheric specialization and side-bias in vertebrates [12]–[13].

However, there are putative disadvantages to lateralization; stereotypical behaviours are by definition easily predicted. Prey with perceptual side-biases should exhibit slower response times to attacks coming from one side versus the other and such a weakness may well be exploited by predators [2], [5], [12]. Behavioural side-bias can also cause developmental asymmetries in the skeleton and musculature [14]. For fast-flying birds, wing asymmetry will reduce flight performance [15]–[17], increase predation-risk [18], and negatively impact fitness [19]. Asymmetrical musculature could likewise be assumed to negatively affect flight performance. Assuming cognitive systems can drive the evolution of behaviours [20], selection should act to reduce the expression of behavioural side-bias when consequences are disadvantageous [6], such as when it will lead to wing or muscular asymmetry. Thus, a behavioural side-bias may be expressed when advantageous and masked when not.

Here, we examine laterality and the expression of side-bias in the broader context of competing constraints. We do this using an aerial hawking, insectivorous bird: the tree swallow, *Tachycineta bicolor*. This species exhibits behavioural side biases in copulatory behaviour on the ground [21] and strong stabilizing selection has been suggested to preserve wing symmetry [22]–[23].

We implement an experimental design in which a bird escapes through a tunnel containing an obstacle varying in size and position. Because fast response times should be favoured during escape, we expected behavioural side-bias might be expressed under our experimental conditions for obstacle-avoidance behaviour. However, given the potential cost of behavioural side-bias in wing and muscular asymmetry (and reduced overall flight performance), we also expected behavioral side-biases might instead be masked. To our knowledge, this is the first study to consider the potential conflict between selection for wing symmetry and selection for side-bias in flying birds.

## Materials and Methods

### (a) Birds, field site and flight tunnel

**Figure 1:**
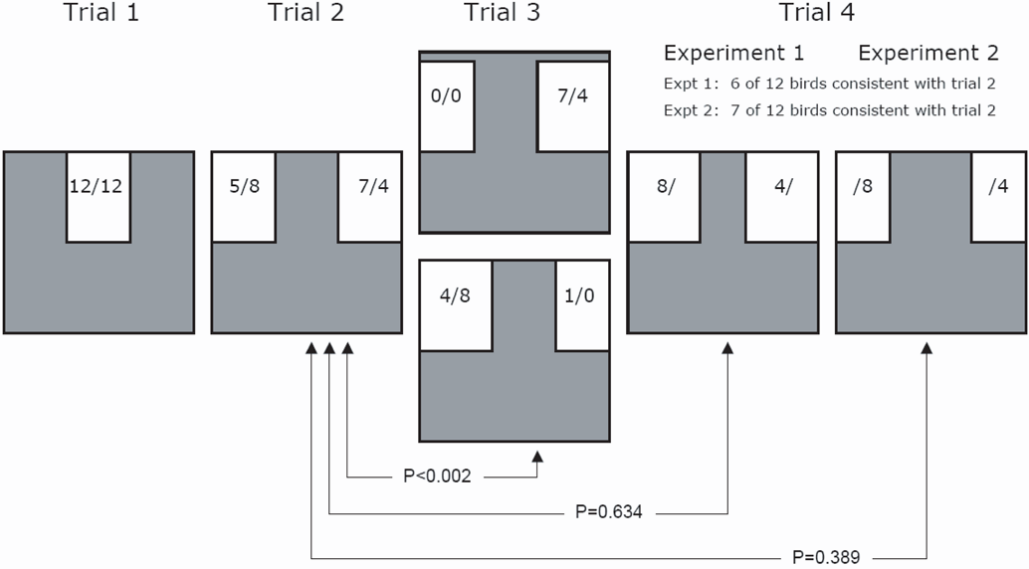
Schematic of the tunnel from the point of view of a bird entering the tunnel. Actual statistics for birds choosing a path is presented in the format x/y, where x represents the 12 birds from experiment one, and y represents the 12 birds from experiment two. In trial three, the side of the optimal choice depended on a bird's choice in trial two. To control for any initial size preference not related to optimality, half of the birds (Experiment 1) were given symmetrical, narrow openings in trial four, while half (Experiment 2) were given symmetrical, wide openings. The comparison of trial two to trial three is a test of optimality, while the comparison of trial two to trial four is a test of side-bias.

Experiments were conducted at the Cornell University Experimental Ponds Facility in Ithaca, New York, U. S. A. (42°30′N, 76°28′W). Twenty-four female tree swallows were captured from their nest boxes during incubation between 24 May and 31 May 2006. Birds were aged by plumage [24], and right and left tarsi and flattened wing lengths were measured (±0.1 mm). During experiments (see below), birds were released individually into an outdoor plywood flight tunnel (1.22 * 1.22 * 9.75 m long) from a lightproof box centred on top of the southwest end. The walls and ceiling of the tunnel were painted matte white and the floor covered with white limestone pebbles in an effort to minimize unintended perceptual asymmetries. The tunnel was illuminated by ten lights distributed equally along the two long walls (for further details of tunnel and study site see [25]. Within the tunnel, 3.22 m from the southwest end, the lower half (h61*w122 cm) was blocked using a light blue Styrofoam sheet (2.5 cm thick). The upper half was partially blocked using sheets 61 cm in height and of various widths (see Figure 1). Each bird flew through the tunnel four times and was caught at the end of the tunnel in a mist net, and was then released at point of capture.

### (b) Experiment 1

Twelve swallows served as subjects in experiment one. These animals did not serve as subjects in experiment two. The purpose of this experiment was to determine whether birds would demonstrate a side-bias while escaping the tunnel or, would make an optimal choice when presented with obstacles within the tunnel: in this case, a larger opening that would be easier to navigate.

First, two light blue h 61 * w 41 cm Styrofoam sections were put into place above the lower sheet flush with the sides of the tunnel, creating a h 61 * w 41 cm centred opening in the upper-half of the obstacle (Figure 1, trial 1). This trial was used to acclimate the bird to the tunnel. In the second trial, a single sheet was positioned such that two equal sized openings (h 61*w 41 cm) exist on either side (Figure 1, trial 2). The bird was released and scored as having used either the right or left opening. In the third trial, this centre section was moved 7.5 cm towards the side the bird had flown through on the previous trial (Figure 1, trial 3). After this trial, the bird was scored as either having made an optimal decision (large opening (h61*w48.5 cm), opposite of side chosen in trial one) or a non-optimal decision (small opening (h61*w33.5 cm), same side as chosen in trial one) and caught. In the fourth and final trial, the off-center upper section used in trial three was removed. A wider section (h61*w56cm) was positioned in the opening, leaving two equal sized openings (h61*w33.5cm) on either side (Figure 1, trial 4). The bird was scored as having used either the same or the opposite opening as used in trial two.

### (c) Experiment 2

Twelve swallows served as subjects in experiment two. The purpose of this experiment was similar to that of the first experiment, but also controlled for a potential confounding variable in the experimental design: that a bird's preference for small or large openings might mask the test of side-bias.

Experiment two was identical to experiment one with the following exception: in the fourth and last trial, rather than use the wide section, a narrow section (h61*w33.5cm), creating two openings the size of the larger opening (h61*w48.5 cm) in trial three was used.

### (d) Statistical analyses

Using the program *R* v. 2.3.1 [26], we ran custom randomization tests to determine if (a) the swallows exhibited a population-level side-bias by testing if the right or left side was chosen on trial two more often than expected by chance (results from two experiments pooled), (b) the optimal side was chosen by individuals more often than expected if both openings had been of equivalent size (results from two experiments pooled; trial 2 versus trial 3), and (c) individuals exhibited a side-bias by testing if the side chosen in trial two was chosen more often than predicted by chance in trial four (tested separately between experiments). The absolute difference in wing and tarsus length between each appendage relative to the average of both appendages was measured. Paired *t*-tests were used to compare the magnitude of asymmetry (i.e. absolute value) between tarsi and wing lengths. *G*-tests of goodness of fit were used to test for a consistent direction of asymmetry (or lack thereof) in the tarsi and wings of individual birds and to test whether such direction of asymmetry in both tarsus and wings was related to side chosen in trial 2 of experiments 1 and 2.

## Results

Seven females were in their second year, nine were >3 years and 8 were adults (>1 yr.) of indeterminate age. All 24 birds flew from the release box to the opposite end of the tunnel for all trials. When the repeated decisions of individual birds are examined, it can be seen that 13 of 24 birds chose the same side in trial 4 as they had in trial 2; both of these trials involved symmetrical openings (Figure 1). Twenty-three of 24 birds chose the larger opening in trial 3, which was always placed opposite to their choice in trial 2 (Figure 1).

We failed to detect evidence of laterality having found no predominance of side bias in trial two of the experiments pooled (500 permutations; actual statistic = 11; p>0.75). The sides chosen in trials two and three were significantly different (500 permutations; actual statistic = 1; p<0.002), indicating that the swallows chose the optimal side for escape in almost all instances. There was also no evidence of functional asymmetry at the individual level; the side chosen between trials two and four did not differ significantly for either experiment one (500 permutations; actual statistic = 6; p = 0.634) or experiment two (500 permutations; actual statistic = 7; p = 0.389).

**Figure 2:**
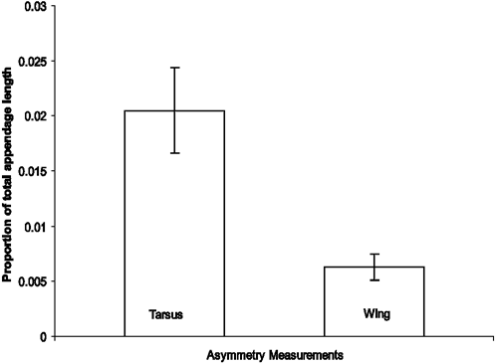
Bar graph showing the means and standard error of asymmetry proportions for both wing and tarsus measurements. The tarsi were significantly more asymmetrical than the wings (t-test, two-tailed, p<0.001).

Tarsus measurements exhibited significantly greater asymmetry than did wing measurements (paired *t*-test; p = 0.002, Figure 2). We found no significant directionality at the population-level in either tarsus or wing asymmetries, and the direction of an individual's side choice in trial 2 was not related to either the direction of that same individual's asymmetry for their wings or tarsi (4 *G*-tests, p>0.3 for all). We also found no significant correlation between tarsus and wing asymmetries Pearson test, r = −.06, p = 0.74).

## Discussion

The results of our experiments suggest that tree swallows do not express functional asymmetries or laterality in obstacle avoidance during escape flight and suggest constraints imposed by selection for morphological symmetry are responsible. Tree swallows chose the larger opening in trials 2 and 3 of both experiments; because tunnel experiments involve collisions with the sides of the tunnel when flying through small openings (DJC and DWW unpublished data), we define the larger opening as the less risky, and optimal, choice. However, as evidenced by the results of trial four in experiment one, the smaller opening was not so small as to prevent birds from flying through it. Tree swallows failed to respond in a consistently lateral manner to symmetrical obstacles in trials 2 and 4, suggesting that these birds do not express functional asymmetry during in-flight escape behaviours at a level detectable by our study.

However, it is possible that with increased overall sample size and a revised experimental protocol that increases trials by individual birds, more subtle expressions of side bias could be found. An exploration of other in-flight behaviours such as predator avoidance, hunting, and conspecific approach could also yield an expression of side bias not seen here in our obstacle-avoidance trial. A third possibility is that swallows approach and evade obstacles in a way that avoids the negative consequences of side bias. Moreover, an individual's behaviour during flight might be a combination of expressed side bias and flight movements that counteract side bias.

Although there are strong links in birds and mammals between brain lateralization and behavioural lateralization (in pigeons, [10]–[11]; in chicks, [9]), functional wing symmetry is critical for anatomical, biomechanical, and energetic features of flight [27]–[28]. In tree swallows, both side-bias [21] and morphological asymmetry in tarsus, bill, and primary feather lengths [29] has been reported. However, repetitious behavioural side-bias can cause morphological asymmetry [14] and conservation of wing symmetry is likely at odds with the expression of laterality and behavioural side-biases in flight. This is supported by comparison of our anatomical and behavioural results. First, asymmetries in individual tarsus lengths varied an order of magnitude more than did those found for individual wing lengths (Figure 2), and, second, these relatively small intra-individual differences in wing length did not predict either initial side choice or side preference in the tunnel (Figure 1).

Ground and aerial hawking foraging strategies are likely subject to different regimes of selective forces. Within flight, different behaviours might also warrant differing expressions of side-bias; predator avoidance, in which the risks are much higher, or conspecific approaches, in which detailed perceptual evaluation is necessary, might carry an expression of side bias. Over evolutionary time, we expect trade-offs between flight performance and hemispheric specialization; for substrate-based activities, we expect similar trade-offs should be less extreme. Our results suggest that hemispheric specialization and the expression of side-biases may not be equally observed under all sensoribehavioural conditions. In birds, chicks [4], [8]–[9], pigeons [9]–[11], Australian magpies [30] and stilts [12] all show behavioural lateralization in at least some substrate-borne activities (e.g., copulation, substrate-borne foraging). Pigeons have clear structural and functional asymmetry of the brain and show behavioural lateralization [31]–[32]. Additionally, they show functional asymmetries of cue use and homing behaviour while flying [11]. However, it is expected that, like tree swallows, this and other species will not show a strongly lateralized motor response to avoid obstacles in flight due to context dependent costs associated with flying. Studies using guppies (*Poeceliid* spp.) and other fish, in similarly designed escape/avoidance trials, report both functional asymmetries and laterality [33]. Side preference varied with the type of obstacle, maintenance of visual contact with the goal, and phylogeny. We suggest that the difference between these results from swimming fish and those from our study using flying tree swallows supports our contention that competing constraints unique to behavioural context and to powered flight lead to the lack of functional asymmetry in our obstacle avoidance trials.

Degree of lateralization and coordination of functional asymmetry at the population-level both vary with gregariousness in fishes [33]. Tree swallows fly in large flocks and roost communally when not breeding [34]; however, in contrast to European starlings, *Sturnus vulgaris* and other birds, flocks do not exhibit any group level polarity of direction [35]. In order to separate the existence of laterality from its expression in flying birds, as well as the strength and context of an effect, further research is required. Limiting visual pathways [11], [31] during a flying obstacle avoidance trial might lead to the expression of a masked trait. In addition, varying the motivation for turning behaviour or the placement and type of visual cues [36] could further define the contexts under which laterality will be expressed or masked. Additionally, future research could try to tightly couple equivalent substrate-born decisions with in-flight decisions; establishing such comparable experiments in quite different contexts would be challenging but worthwhile.

Research into laterality often uses side-bias as a means of detecting hemispheric specialization [12]. However, in situations where expression of laterality is disadvantageous, masking could occur. We suggest that future research efforts consider information acquisition and decision-making under a broad range of ecological contexts and contingencies in light of the potential trade-offs between the behavioural and morphological outcomes of these processes and the pervasive vertebrate hemispheric specialization and expressed or masked side-biases.
